# Pharmacokinetics and local tissue response to local instillation of vocacapsaicin, a novel capsaicin prodrug, in rat and rabbit osteotomy models

**DOI:** 10.1002/jor.25271

**Published:** 2022-02-06

**Authors:** Tarra Knotts, Kirsten Mease, Lakshmi Sangameswaran, Melanie Felx, Susan Kramer, John Donovan

**Affiliations:** ^1^ Concentric Analgesics San Francisco California USA; ^2^ ToxStrategies, Inc. Cary North Carolina USA; ^3^ Independent Consultant San Jose California USA; ^4^ Charles River Laboratories Montreal Quebec Canada

**Keywords:** analgesia, capsaicin, osteotomy, postoperative pain, prodrug

## Abstract

Vocacapsaicin is a novel prodrug of trans‐capsaicin (trans‐8‐methyl‐*N*‐vanillyl‐6‐nonenamide) being developed as a nonopioid, long‐lasting, site‐specific treatment for postsurgical pain management. The objective of these studies was to examine the safety and tolerability of vocacapsaicin in an osteotomy model in two animal species and to evaluate bone healing parameters. Rats undergoing unilateral femoral osteotomy received a single perioperative administration (by instillation) of vocacapsaicin (vehicle, 0.15, 0.3, and 0.6 mg/kg). Rabbits undergoing unilateral ulnar osteotomy received a single perioperative administration (by infiltration and instillation) of vocacapsaicin (vehicle, 0.256 and 0.52 mg) alone or in combination with 0.5% ropivacaine. Clinical signs, body weights, food consumption, radiography, histopathologic examinations, ex vivo bone mineral density measurements (rats only), and biomechanical testing were evaluated at 4 and 8 weeks in rats and at 2 and 10 weeks in rabbits. Plasma samples were also collected in rabbits. There were no vocacapsaicin‐related effects on mortality, clinical observations, body weight, or food consumption in either species. Systemic exposure to vocacapsaicin and its metabolites, including capsaicin, was transient. In rats, vocacapsaicin was devoid of deleterious effects on bone healing parameters, and there was a trend for enhanced bone healing in rats treated with the mid‐dose. In rabbits, vocacapsaicin administered alone or in combination with ropivacaine did not adversely affect bone healing parameters. In conclusion, a single perioperative administration of vocacapsaicin in unilateral osteotomy models was well tolerated, locally and systemically, supporting its continued development as a novel, nonopioid treatment for postsurgical pain management.

## 1 INTRODUCTION

Enhanced recovery protocols have improved postsurgical outcomes in a wide array of surgeries. A cornerstone of this approach is multimodal analgesia, which utilizes a range of mechanistically distinct pharmacological treatments to inhibit nociceptive processing at multiple levels of the neuroaxis.^1^ By using various combinations of acetaminophen, nonsteroidal anti‐inflammatory drug, gabapentinoids, and local anesthetics, it is possible to decrease postsurgical opioid requirements, and, in turn, opioid‐related adverse events and risk of persistent opioid use in the outpatient setting.^2^ Opioid‐sparing strategies have been shown to reduce acute postoperative pain, the risk of developing chronic pain, length of hospital stay, and per patient costs.^3^, ^4^, ^5^, ^6^ Despite these advances, up to 75% of patients report significant pain in the postsurgical setting.^7^, ^8^ There remains an unmet need to for enhanced nonopioid modalities for the management of acute postoperative pain.

**Figure 1:**
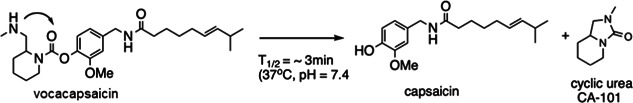
Mechanism of conversion of vocacapsaicin to capsaicin and CA‐101

Vocacapsaicin, formerly designated as CA‐008 HCl, is a novel prodrug of trans‐capsaicin (trans‐8‐methyl‐*N*‐vanillyl‐6‐nonenamide) being developed as a nonopioid, site‐specific treatment for postsurgical pain management. It is designed to provide long‐lasting (at least 96 h) postsurgical analgesia following a single intraoperative administration. The free base form of vocacapsaicin rapidly cyclizes under physiological conditions to yield active capsaicin and CA‐101 (a cyclic urea) as the sole conversion products (Figure 1). CA‐101 has been evaluated in vitro for biological activity and was demonstrated to be inactive (data on file, Concentric Analgesics).

Once released from vocacapsaicin, capsaicin produces agonist activity at TRPV1 receptors located on primary afferent C‐fibers.^9^, ^10^, ^11^ This neuronal excitation initially causes a painful, burning sensation; however, with continued depolarization, the nociceptors become reversibly desensitized, leading to durable analgesia.^12^ Capsaicin has been used for decades as a topical analgesic, but its utility for other routes of administration has been limited by its poor water solubility.

Prodrugs afford one approach for altering the physicochemical properties of existing molecules to enhance their delivery to the desired tissue.^13^ Vocacapsaicin was developed to improve upon the solubility profile of capsaicin and allow for an aqueous formulation that could be simply administered in the wound site to achieve a long‐lasting analgesic effect. Vocacapsaicin has little or no pharmacologic activity as the intact prodrug and is rapidly converted to capsaicin. As vocacapsaicin is administered and designed to release capsaicin rapidly at the surgical site, in contrast to historical topical uses, it is important to examine its overall safety and tolerability profile, including any systemic exposure and evaluation of any effects on healing at the surgical site. The objective of these studies was to examine the safety and tolerability of vocacapsaicin in an osteotomy model in two animal species (rats and rabbits), and to evaluate radiographic, histopathologic, and biomechanical bone healing parameters in both species. While analgesia was not formally measured in these studies, concentrations of vocacapsaicin were equivalent to or higher than those associated with postsurgical analgesia in randomized, controlled clinical trials in patients following bunionectomy and total knee arthroplasty.^14^, ^15^

## 2 METHODS

### 2.1 Animals

#### 2.1.1 Rat (femoral osteotomy model)

Female Sprague–Dawley rats (Charles River Canada) 13–14 weeks of age and weighing 230–330 g were individually housed in a room maintained at 19–25°C on a 12‐h light/dark cycle. Food and water were available ad libitum. The study plan was reviewed and approved by Charles River MTL Institutional Animal Care and Use Committee (IACUC).

Rats were anesthetized with isoflurane gas and prepared for surgery. An incision was made on the lateral aspect of the right femur. The fascia, separating the tensor fascia lata and biceps femoris muscles, was identified and incised. The vastus lateralis muscle was elevated off the lateral border of the femur, from the greater trochanter to the lateral femoral trochanter condyle, with care to preserve the overlying periosteum. A fixation kit that included a connecting polyether ether ketone plate and four metallic screws was used. An osteotomy was performed at the midshaft using an oscillating saw. The osteotomy site was thoroughly irrigated with sterile fluids during the osteotomy to avoid any microdamage caused by heat. A cerclage wire was used to stabilize the bone and the site was irrigated with saline to remove bone debris. The soft tissues were meticulously closed in layers. At least 30 min before surgery, subcutaneous injections of buprenorphine (0.1 mg/kg), meloxicam (2 mg/kg), and trimethoprim sulfa (30 mg/kg) were administered to each rat. Following surgery, three additional injections of buprenorphine and trimethoprim sulfa were administered at 12‐h intervals (for a total of 4 injections), and two additional injections of meloxicam were administered at approximate 24‐h intervals (for a total of 3 injections). Additional doses of buprenorphine and meloxicam could be administered thereafter at the discretion of the study director.

Within 2 days of arriving at the animal facility and before surgery, rats were assigned to dosing groups in a blinded process by a stratified randomization scheme designed to achieve similar body weights among groups. Rats were assigned to one of four dosing groups (*n* = 35–36/group): 0 (vehicle control—saline for Injection, USP), 0.15, 0.3, or 0.6 mg/kg vocacapsaicin. Each dosing group was comprised of a 4‐week and an 8‐week subset of animals (*n* = 17–18/subset) (Table 1). Animals in poor health or at extremes of body weight range were not assigned to groups. Immediately after surgery, vehicle or vocacapsaicin was administered once (in a volume of 0.5 ml/kg), before wound closure, by instillation directly on the surgical site area such that there was exposure of the test material to the cut tissues including the bone in the surgery site. The first day of dosing was designated as Day 1. The effect of vocacapsaicin on bone healing was evaluated at 4 (Day 29) and 8 weeks (Day 57) after osteotomy. Animals were euthanized either 4 or 8 weeks postsurgery via exsanguination from the abdominal aorta under isoflurane anesthesia.

**Table 1:** Dosing for rat unilateral femoral osteotomy model

Group No.^a^	Test material	Dose (mg/kg)	Concentration (mg/ml)	Dose volume (ml/kg)	No. of animals^b^	
					4‐Week subset	8‐Week subset
					Female	Female
1	Saline	0	0	0.5	18	17^c^
2	Vocacapsaicin	0.15	0.3	0.5	18	18
3	Vocacapsaicin	0.30	0.6	0.5	18	18
4	Vocacapsaicin	0.6	1.2	0.5	18	18

^a^ Rats were assigned to dosing groups using a stratified randomization scheme designed to achieve similar mean body weights.

^b^ Several animals were replaced by spare animals (as established a priori) due to radiographic observations (e.g., incomplete fracture), death during surgery, or poor condition following surgery.

^c^ One animal was euthanized during surgery and not replaced.

Mortality and moribundity checks were performed twice daily and cage side observations were performed once daily throughout the study. Detailed clinical observations were performed on Days 4 and 7 postsurgery, and then weekly or the remainder of the study. Food was available ad libitum throughout the study. Food consumption and body weight were measured weekly starting the week before surgery.

#### 2.1.2 Rabbit (ulnar osteotomy model)

Male and female New Zealand White rabbits (Covance Research Products Inc.) 4–5 months of age were individually housed in a room maintained at 16–22°C on a 12‐h light/dark cycle. At initiation of dosing, males weighed 2.2–3.1 kg and females weighed 2.0–3.2 kg. All animals received 120 g per day of a standard commercial laboratory diet (PMI Certified Rabbit 5325: PMI Nutrition International Inc.) and water was available ad libitum. The study plan was reviewed and approved by Charles River MTL IACUC.

Rabbits were anesthetized with ketamine and dexmedetomidine before surgical preparation and were maintained under isoflurane anesthesia for the entire surgery. An incision of approximately 4 cm in length was performed on the lateral aspect of the distal ulna of the right forelimb and the ulna was exposed extraperiosteally. The periosteum was cut longitudinally and detached from the bone along the incision. A cut the width of the saw blade was performed in the mid‐ulna using a pendular saw. The soft tissues were meticulously closed in layers. Each animal received subcutaneous injections of trimethoprim‐sulfa 24% (30 mg/kg), buprenorphine (0.05 mg/kg), and carprofen (4 mg/kg) 30 min before surgery. Following surgery, additional doses of trimethoprim‐sulfa 24% (q12 h for 2 days), buprenorphine SR (0.1 mg/kg, 2 injections, 3 days apart), and carprofen (qd for 7 days) were administered.

Vehicle, vocacapsaicin, ropivacaine, and vocacapsaicin/ropivacaine were administered with a syringe needle on the day of surgery (Day 1) as described in Table 2. For Groups 1–3, Vehicle (Group 1) or vocacapsaicin (Groups 2 and 3) was administered by a combination of instillation (0.1 ml) before wound closure and infiltration (0.7 ml) around the surgical site. For Groups 4–6, 0.8 ml of 0.5% ropivacaine was administered by infiltration at the start of the surgery. For Groups 5 and 6, after an approximate 20‐min interval, vocacapsaicin was administered by instillation (0.1 ml) at the surgical site before wound closure and by infiltration (0.7 ml) around the surgical site. The total volume of the sequential dosing of ropivacaine and vocacapsaicin was 1.6 ml. Pharmacokinetic (PK) characteristics of vocacapsaicin and its major metabolites, CA‐101 and capsaicin, were determined after dosing. Bone healing parameters were evaluated at 2 weeks (Day 14) and 10 weeks (Day 70) after osteotomy.

**Table 2:** Dosing for rabbit unilateral ulnar osteotomy model

Group No.^a^	Test material	Dose of vocacapsaicin^b^ (mg/dose)	Volume (ml/site)	Concentration (mg/ml)	Number of animals^c^			
					2‐Week subset^d^		10‐Week subset^e^	
					M	F	M	F
1	Vehicle	0	0.8/–	0/–	3	3	10	10
2	Vocacapsaicin	0.256	0.8/–	0.32/–	3	3	10	10
3	Vocacapsaicin	0.520	0.8/–	0.65/–	3	3	10	10
4	Ropivacaine 0.5%	0	–/0.8	–/5	3	3	10	10
5	Vocacapsaicin/ropivacaine 0.5%	0.256	0.8/0.8	0.32/5	3	3	10	10
6	Vocacapsaicin/ropivacaine 0.5%	0.520	0.8/0.8	0.65/5	3	3	10	10

*Note*: – represents not applicable.

Abbreviations: F, females; M, males.

^a^ Rabbits were assigned to dosing groups using a stratified randomization scheme designed to achieve similar mean body weights.

^b^ On the basis of a 2.5 kg body weight.

^c^ Several animals were replaced by spare animals (as established a priori) due to radiographic observations (e.g., incomplete fracture) or poor condition following surgery.

^d^ Scheduled for termination on Week 2 for histopathological evaluation.

^e^ Scheduled for termination on Week 10, 2 animals/sex/group for histopathological evaluation and 8/sex/group for biomechanical testing.

Mortality and moribundity checks were performed twice daily throughout the study. Detailed clinical observations were performed before treatment, daily from Day 1 to Day 10, and weekly thereafter. Food consumption was measured daily from Day −1 to the end of the study. Body weights were measured weekly starting at Week −3.

#### 2.1.3 Bioanalysis and PK evaluation (rabbits only)

Blood (1 ml target volume) from rabbits was collected from an appropriate auricular vessel before dosing and at 0.25, 0.5, 1, 2, 4, 6, 8, 12, 24, 48, and 96 h postdose. Samples were mixed gently, and placed on crushed wet ice, and centrifuged as per standard procedures within 30 min after collection. The resultant plasma was separated into two approximately equal aliquots. Plasma samples were frozen immediately over dry ice and transferred to a freezer maintained at −20°C within 90 min from the time of blood collection. Plasma samples were analyzed (Worldwide Clinical Trials) by liquid chromatography with tandem mass spectrometry for concentration of CA‐008, CA‐101, and capsaicin using a validated analytical procedure.

PK parameters were estimated for CA‐008, CA‐101, and capsaicin using noncompartmental methods with Phoenix PK software. The following parameters were calculated for each analyte: time after dosing at which the maximum concentration was observed (*T*_max_), the maximum observed concentration (C_max_), the area under the concentration versus time curve from dosing to time of last quantifiable concentration (AUC_0‐*t*_), AUC_0‐*t*_/dose, C_max_/dose, and half‐life (*t*_1/2_).

#### 2.1.4 Radiography

##### Rats

Radiographs of the femur (mediolateral view) were performed under isoflurane anesthesia on the day of surgery using Dragon Digital‐X‐ray System (Raytech Diagnostics) for all animals, at Week 4 for animals in the 4‐ and 8‐week subsets using Faxitron (Faxitron Bioptics LLC), and at Week 8 for animals in the 8‐week subset. Radiographs were evaluated semiquantitatively to determine healing. The radiographs were graded for the extent of callus formation and bone union according to three categories, adapted and modified from An et al.^16^: periosteal and endosteal reaction (range: 0 [none]–3 [marked bridging across the osteotomy]), callus opacity (range: 0 [no evidence of mineralization]–3 [confluent with cortices, uniform]), and cortical remodeling and bridging (range: 0 [no apparent remodeling])–4 [complete cortical union]).

##### Rabbits

Radiographs of the ulna (mediolateral view) were performed under isoflurane anesthesia using MTM Digital X‐ray System (Minnetonka Medical Technology, Inc.) on the day of surgery and at Weeks 6, 8, and 10, and evaluated semiquantitatively to determine healing. The radiographs were graded for the extent of callus formation and bone union as described above for rats.

#### 2.1.5 Histopathology

##### Rats

The right femur, popliteal, and iliac lymph node from eight animals per dose group from the 4‐ and 8‐week subsets were embedded in paraffin, sectioned, and mounted on glass slides. For the femur, one section was stained with hematoxylin and eosin and another section was stained with Safranin O. Lymph nodes were stained with hematoxylin and eosin. Histopathological evaluation was performed by a board‐certified veterinary pathologist. Microscopic evaluation was performed to assess healing of the osteotomy site, using a semiquantitative grading scheme in which callus maturity was graded on a 1 (minimal)–5 (severe) scale.

##### Rabbits

For the histopathological population, the right ulna bone and the surrounding soft tissues were embedded in paraffin, sectioned, mounted on glass slides, and stained with hematoxylin and eosin. Histopathological evaluation was performed by a board‐certified veterinary pathologist.

#### 2.1.6 Ex vivo bone densitometry (rats only)

Peripheral quantitative computed tomography (pQCT) scans of the osteotomy site were performed using an XCT Research SA bone scanner (Stratec Medizintechnik). The first slice was performed proximally and adjacent to the osteotomy site and four subsequent slices were then acquired distally to image as much of the osteotomy site as possible. The three sequential slices determined to most accurately span the osteotomy site were selected for analysis. The area (mm^2^), bone mineral content (mg/mm), and bone mineral density (mg/cm^3^) were reported for the total osteotomy site, the mature callus area, and the immature callus area, using averaged values from analyses of the three approved slices.

#### 2.1.7 Biomechanical testing

##### Rats

A 4‐point bending test was performed on the right femur of up to 10 terminally euthanized animals per dosing group, from the 4‐ and 8‐week subsets, using the Bose Electroforce® 3300 System (TA Instruments). The femur was positioned on the lower fixture in the most stable position. The upper fixture was positioned in a manner that the mid callus was in the middle of the upper and lower contact points. Testing parameters included: peak load (N), stiffness (N/mm), and work to failure (area under the curve, N·mm). The bone data and associated report were peer reviewed by an external expert.

##### Rabbits

A 4‐point bending test was performed on all 10‐week subset animals selected for biomechanical testing, using the MTS 858 Mini Bionix Servohydraulic Test System (MTS Systems Corporation). The ulna was carefully separated from the radius using a diamond coated water‐cooled and low‐speed saw before biomechanical testing. The cranial aspect of the ulna was positioned on the lower fixture in the most stable position. Upper fixture was positioned in a manner that the midcallus was in the middle of the upper and lower contacts points. Testing parameters included: peak load (N), stiffness (N/mm), and work to failure (area under the curve, N∙mm).

#### 2.1.8 Drugs

##### Rat study

A stock solution of vocacapsaicin (2.4 mg/ml) at pH 3 was prepared and used to prepare individual dosing solutions. The appropriate amount of vocacapsaicin item was dissolved in pH 3.0 saline to meet necessary volume requirements, and the pH of the resulting solution was adjusted to 4.5 using 0.01 N NaOH. Vehicle control was saline for injection, USP.

##### Rabbit study

Vocacapsaicin was dissolved in citrate buffer and diluted with saline, with a final pH of 3.6– 3.8. Ropivacaine (0.5%, 5.0 mg/ml) was filtered, aliquoted, and then dispensed as received on the day of dosing for administration. Vehicle control consisted of 0.5 mM citrate buffer and 0.4 mg/ml mannitol, in saline, with a final pH of 3.5–3.8.

#### 2.1.9 Statistical analyses

Descriptive statistics of mean and standard deviation (or %CV or SE when deemed appropriate) were reported whenever possible. Inferential analyses were conducted for body weight, bone biomechanics, and densitometry (rats only) using a one‐way analysis of variance or the Kruskal–Wallis test when group variances were not homogenous. If significance was achieved, then pairwise comparisons were conducted using a two‐sided *t*‐test or Wilcoxon rank sum test, respectively. In the rat study, pairwise comparisons between each dose of vocacapsaicin versus placebo were conducted. In the rabbit study, pairwise comparisons of interest included: vocacapsaicin (both doses) versus vehicle; and vocacapsaicin (both doses) + 0.5% ropivacaine versus 0.5% ropivacaine alone. Adjustments for multiplicity were made based on the square root of the number of pairwise comparisons. All statistical tests were conducted at the 5% significance level.

## 3 RESULTS

### 3.1 Rat osteotomy

#### 3.1.1 Mortality, clinical signs, body weights, and food consumption

Across the dose range tested, 0.15 mg/kg (0.3 mg/ml) to 0.6 mg/kg (1.2 mg/ml), there were no vocacapsaicin‐related effects on mortality, clinical signs, body weights, or food consumption. During the first week of the study, several animals lost weight, an effect attributed to the surgery. Correspondingly, food intake was decreased during the first week of the study and subsequently increased during the second week and remained constant for the remainder of the study.

#### 3.1.2 In vivo radiographic evaluation

**Figure 2:**
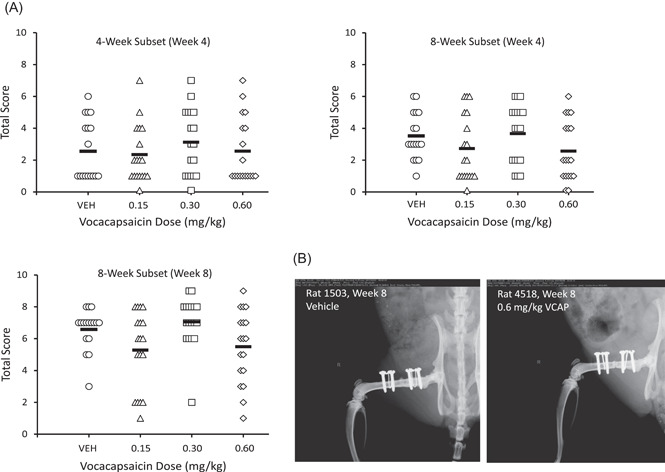
(A) Radiographic total healing score in 4‐ and 8‐week subsets of rats (*n* = 17–18/subset/treatment group) treated with vehicle (VEH) or vocacapsaicin. Each point represents data from an individual animal and the solid black line represents the mean of each treatment group. (B) Radiographs for representative rats treated with vehicle or 0.6 mg/kg vocacapsaicin

At Week 4 in both the 4‐ and 8‐week subsets of animals, there was a trend for increases in healing scores in animals dosed with 0.3 mg/kg (0.6 mg/ml) vocacapsaicin compared to vehicle controls (Figure 2). Healing scores in animals dosed with 0.15 mg/kg (0.3 mg/ml) and 0.6 mg/kg (1.2 mg/ml) vocacapsaicin were comparable to vehicle controls. At Week 8, there were no vocacapsaicin‐related changes in radiographic healing scores in animals administered any dose of vocacapsaicin compared to vehicle controls. All groups were demonstrating normal healing (Figure 2). Radiographs are provided for representative rats treated with vehicle or 0.6 mg/kg (1.2 mg/ml) vocacapsaicin.

#### 3.1.3 Histopathology

Consistent with the radiographic findings, in the 4‐week subset, an increased number of rats dosed with 0.3 mg/kg (0.6 mg/ml) vocacapsaicin had mature or nearly mature calluses (calluses of either Grade 4 or 5) in comparison to vehicle controls (Table 3). At the high dose of 0.6 mg/kg (1.2 mg/ml), a marginal increase in the number of rats with mature or nearly mature calluses was also observed. In the 8‐week subset, the incidence of mature or nearly mature calluses was similar between vocacapsaicin‐treated rats and vehicle controls. There was considerable variability in the extent of callus formation within treatment groups. For example, in the 8‐week subset of rats treated with 0.15 mg/kg vocacapsaicin, four of the rats had minimal callus formation (score of 1) and the other four rats had severe callus maturity (score of 5).

**Table 3:** Summary of microscopic findings in 4‐ and 8‐week subsets of rats treated with vehicle or vocacapsaicin

	**4‐Week subset**				**8‐Week subset**			
Group	1	2	3	4	1	2	3	4
Dose (mg/kg)	0	0.15	0.3	0.6	0	0.15	0.3	0.6
Concentration (mg/ml)	0	0.30	0.6	1.2	0	0.30	0.6	1.2
No animals	8	8	8	8	8	8	8	8
Bone femur (no examined)	8	8	8	8	8	8	8	8
Callus maturity								
Minimal (1)	1	1	1	0	0	4	1	0
Mild (2)	2	3	1	2	0	0	1	0
Moderate (3)	3	3	1	3	1	0	1	3
Marked (4)	1	0	2	0	1	0	0	1
Severe (5)	1	1	3	3	6	4	5	4

In both the 4‐ and 8‐week subsets, the nature and microscopic features of these calluses, which were generally formed from intramembranous ossification, were similar in vocacapsaicin‐treated and vehicle‐treated animals.

#### 3.1.4 Ex vivo bone densitometry

At Week 4 and Week 8, there were no vocacapsaicin‐related changes in femur pQCT parameters in animals treated with any dose of vocacapsaicin relative to vehicle (Table 4). Non‐dose‐dependent differences compared to controls (most notably at the low dose of 0.15 [0.3 mg/ml] and high dose of 0.6 mg/kg [1.2 mg/ml]) were generally due to one or two individuals that skewed mean values and were, therefore, attributed to interindividual variability.

**Table 4:** Summary of bone densitometry values (mean [SD]) by peripheral quantitative computed tomography in 4‐ and 8‐week subsets of rats treated with vehicle or vocacapsaicin

	4‐Week subset				8‐Week subset			
Group	1	2	3	4	1	2	3	4
Dose group (mg/kg)	0	0.15	0.3	0.6	0	0.15	0.3	0.6
Concentration (mg/ml)	0	0.30	0.6	1.2	0	0.30	0.6	1.2
No animals	10	10	10	10	9	9	9	9
Total								
Area (mm^2^)	13.4 (2.2)	12.6 (2.1)	12.9 (1.3)	12.45 (1.6)	13.7 (2.5)	12.6 (2.9)	13.6 (2.1)	11.7 (3.8)
BMC	9.8 (1.8)	10.0 (2.6)	10.1 (2.2)	9.5 (1.7)	12.6 (2.4)	11.4 (2.9)	12.3 (1.3)	10.6 (4.5)
BMD (mg/cm^2^)	735.6 (77.3)	781.3 (111.2)	777.5 (127.1)	764.3 (80.9)	923.2 (86.3)	900.6 (96.0)	915.5 (100.6)	847.0 (218.8)
Mature callus								
Area (mm^2^)	8.9 (1.8)	8.8 (2.4)	8.9 (2.1)	8.7 (1.9)	10.7 (1.7)	9.7 (2.2)	10.4 (1.4)	9.1 (3.9)
BMC	8.4 (1.7)	8.7 (2.7)	8.9 (2.5)	8.1 (1.9)	11.6 (2.3)	10.4 (2.8)	11.3 (1.2)	9.7 (4.5)
BMD (mg/cm^2^)	946.7 (53.7)	967.0 (80.6)	974.0 (112.9)	928.1 (47.0)	1075.5 (89.1)	1052.8 (93.5)	1092.6 (43.9)	987.5 (213.8)
Immature callus								
Area (mm^2^)	4.5 (1.5)	3.7 (1.1)	4.0 (1.2)	3.8 (1.2)	2.9 (1.2)	2.8 (1.6)	3.2 (1.9)	2.7 (1.0)
BMC	1.5 (0.4)	1.3 (0.3)	1.3 (0.3)	1.4 (0.4)	1.0 (0.3)	1.0 (0.5)	0.9 (0.4)	0.9 (0.3)
BMD (mg/cm^2^)	333.9 (60.4)	356.1 (58.8)	325.5 (48.4)	373.4 (26.7)	358.8 (52.5)	369.2 (46.9)	330.4 (61.2)	358.2 (63.6)

Abbreviations: BMC, bone mineral content; BMD, bone mineral density.

#### 3.1.5 Biomechanical testing

**Figure 3:**
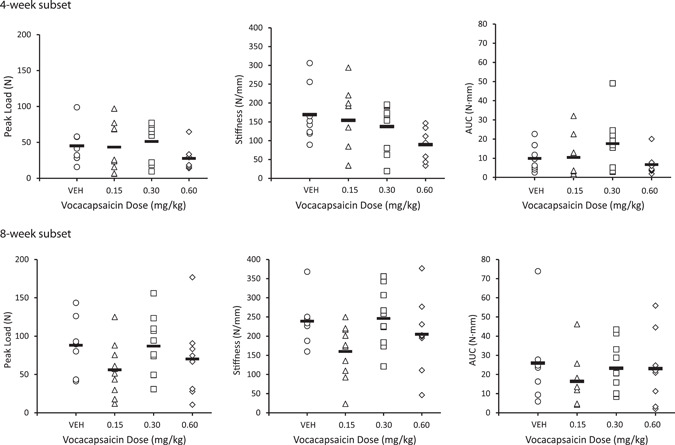
Biomechanical evaluation (4‐point bending) in 4‐week (top panels) and 8‐week (bottom panels) subsets of rats (*n* = 8–10/subset/treatment group) treated with vehicle (VEH) or vocacapsaicin. Testing parameters included: peak load (Newtons [N]), stiffness (N/mm), and work to failure (area under the curve [AUC], N · mm), expressed as mean (SE)

At Week 4, there was a trend for an increase in AUC (+78%) for the femur in 4‐point bending in animals administered 0.3 mg/kg (0.6 mg/ml) compared to controls, suggestive of increased callus strength (Figure 3). There were no vocacapsaicin‐related changes in extrinsic parameters (peak load, stiffness, and AUC) in animals given 0.15 and 0.6 mg/kg. Non‐dose‐dependent differences observed in mean values, most notably at 0.6 mg/kg, were attributed to individual animal variation and were, therefore, considered unrelated to vocacapsaicin treatment.

At Week 8, there were no vocacapsaicin‐related changes in extrinsic parameters in animals

administered any dose of vocacapsaicin compared to controls (Figure 3).

### 3.2 Rabbit osteotomy

#### 3.2.1 Mortality, clinical signs, body weights, and food consumption

Across the two doses tested, 0.256 (0.32 mg/ml) and 0.52 mg (0.65 mg/ml), there were no vocacapsaicin‐related effects on mortality, clinical signs, body weights, or food consumption, with or without 0.5% ropivacaine administration. Two male rabbits were euthanized in an unscheduled manner. One male rabbit administered vocacapsaicin 0.256 mg + 0.5% ropivacaine was euthanized on Day 39 because of the limited usage of the right forelimb, which had persisted since Day 2. At the macroscopic examination, the proximal right ulna was bent at the osteotomy site. The exact contributory effect vocacapsaicin and ropivacaine remain uncertain due to the expected individual variability in the healing process in this model. A second male administered vocacapsaicin 0.256 mg was euthanized on Day 29 because of large cutaneous ulceration on the scapular region, which was unrelated to vocacapsaicin administration.

#### 3.2.2 Pharmacokinetics

**Figure 4:**
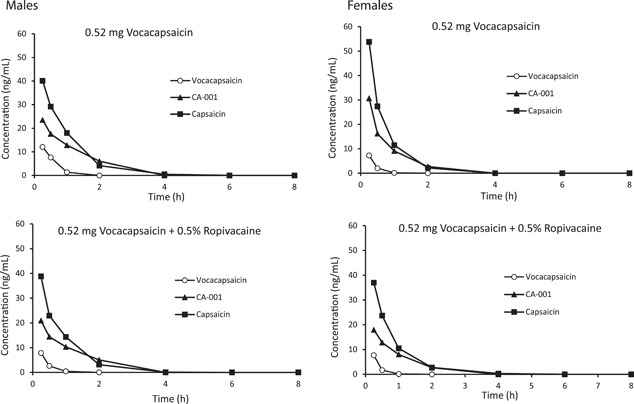
Mean plasma concentrations of vocacapsaicin, CA‐101, and capsaicin following infiltration and instillation of 0.52 mg/kg vocacapsaicin ± 0.5% ropivacaine in male (left panels) and female (right panels) rabbits (*n* = 3/sex/treatment group)

In males and females, systemic exposure to CA‐008 was transient, with *T*_max_ observed at the first sampling time (15 min) and concentrations decreasing below the limit of quantitation by 2 h postdose (Figure 4). Similarly, for CA‐101 and capsaicin in both males and females, mean *T*_max_ was observed at the first sampling time and concentrations were below the limit of quantitation by 4 h postdose. Derived PK parameters for all three analytes indicate dose‐dependent increases in exposure that were generally linear (Table 5). For all three analytes, exposure was slightly lower when administered with ropivacaine. There were no sex differences in exposure.

**Table 5:** Plasma PK parameters (mean [SD]) of vocacapsaicin (VCAP), CA‐101, and capsaicin following infiltration and instillation of 0.52 mg/kg vocacapsaicin alone or in combination with 0.5% ropivacaine in male and female rabbits (*n* = 3/sex/treatment group)

Sex		Vocacapsaicin		CA‐101		Capsaicin	
		C_max_	AUC	C_max_	AUC	C_max_	AUC
Male	VCAP alone	12.1 (4.6)	6.2 (3.7)	25.5 (6.5)	25.1 (6.7)	43.5 (5.1)	41.1 (18.1)
	VCAP + ropivacaine	7.8 (1.4)	3.1 (0.3)	20.9 (4.4)	20.9 (1.7)	38.8 (10.7)	32.0 (6.0)
Female	VCAP alone	7.3 (0.9)	2.6 (0.1)	30.7 (2.7)	22.0 (1.9)	53.8 (1.8)	33.5 (0.9)
	VCAP + ropivacaine	7.7 (1.0)	2.5 (0.5)	18.0 (1.8)	15.9 (5.9)	37.0 (0.5)	29.9 (6.2)

Abbreviations: AUC, area under the curve; C_max_, maximum observed concentration; PK, pharmacokinetic.

#### 3.2.3 In vivo radiographic evaluation

**Figure 5:**
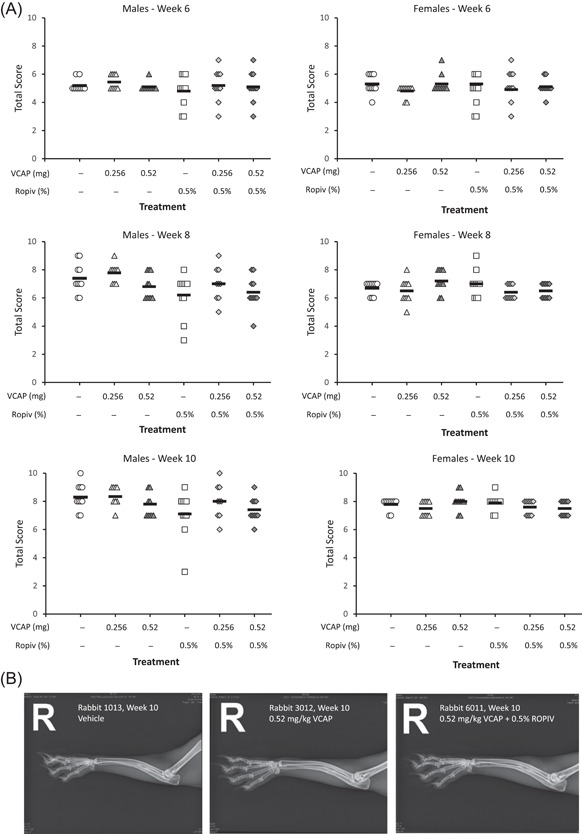
(A) Radiographic total healing score at Week 6, 8, and 10 in male (left panels) and female (right panels) rabbits (*n* = 9–10/sex/treatment group) treated with vehicle or vocacapsaicin ± 0.5% ropivacaine. Each point represents data from an individual animal and the solid black line represents the mean of each treatment group. (B) Radiographs for representative rabbits treated with vehicle, 0.52 mg vocacapsaicin (VCAP), and 0.52 mg vocacapsaicin +0.5% ropivacaine (ROPIV)

Radiographic healing scores increased over the course of the study in all treatment groups. At Weeks 6, 8, and 10, there were no vocacapsaicin‐related differences in radiographic healing score in males or females administered vocacapsaicin alone or in combination with ropivacaine, compared to their respective vehicle controls (Figure 5). Radiographs are provided in representative rabbits treated with vehicle, 0.52 mg vocacapsaicin, or 0.52 mg vocacapsaicin + 0.5% ropivacaine.

#### 3.2.4 Gross pathology and histopathology

No vocacapsaicin‐related gross or microscopic findings were noted in the 2‐ or 10‐week subsets of animals. At 2 weeks postsurgery, the callus was variously sized and composed of fibrous tissue, cartilage, and bone but was comparable among groups, including controls. Complete fusion (union) of the osteotomy sites did not occur in any of the animals at this time point; however, a callus was present for each animal, as expected for this model. At 10 weeks postsurgery, all animals evidenced complete union at the osteotomy site as well as decreased size of the callus (composed exclusively of bone) compared to those observed 2 weeks postsurgery. The incidence and severity of the callus formation were comparable between control animals and animals administered vocacapsaicin with or without ropivacaine.

#### 3.2.5 Biomechanical testing

**Figure 6:**
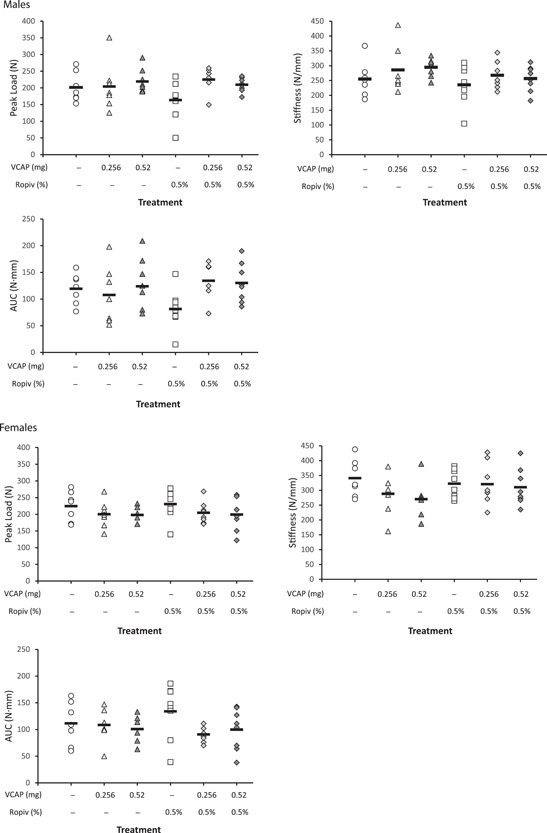
Biomechanical evaluation (4‐point bending at Week 10) in male (top panels) and female (bottom panels) rabbits (*n* = 7–8/sex/treatment group) treated with vehicle or vocacapsaicin +0.5% ropivacaine. Testing parameters included: peak load (Newtons [N]), stiffness (N/mm), and work to failure (area under the curve [AUC], N · mm), expressed as mean (SE). ROPIV, ropivacaine; VCAP, vocacapsaicin

At Week 10, there were no vocacapsaicin‐related differences in ulnar 4‐point bending (peak load, stiffness, and AUC) in males or females administered any dose of vocacapsaicin, alone or in combination with ropivacaine, relative to their respective controls (Figure 6).

## 4 DISCUSSION

The objective of these studies was to examine the safety and tolerability of a novel prodrug of trans‐capsaicin, vocacapsaicin, in an osteotomy model in rats and rabbits, and to evaluate radiographic, histopathologic, and biomechanical bone healing parameters in both species. Vocacapsaicin was administered on a single occasion by instillation (rats) or a combination of instillation and infiltration around the surgical site (rabbits). There were no effects of vocacapsaicin on mortality, clinical signs, body weights, or food consumption when tested up to human equivalent doses of 5.4 and 9.3 mg (free base) in rats and rabbits, respectively.

In addition to the lack of adverse clinical signs, there were no deleterious effects of vocacapsaicin on bone healing after osteotomy surgery in rats and rabbits as assessed macroscopically or microscopically and by radiography, densitometry (rats only), and biomechanical testing. This was true in rats assessed 4 and 8 weeks following a single perioperative administration of vocacapsaicin and in rabbits assessed 2 and 10 weeks following a single perioperative administration of vocacapsaicin, with or without ropivacaine. While there was considerable variability within treatment groups across bone healing parameters in both species, there was no discernable dose–response relationship following vocacapsaicin treatment for any parameter. The lack of effect on bone healing with vocacapsaicin is concordant with preclinical observations with an investigational, purified formulation of capsaicin in bone and wound healing models.^17^, ^18^ In rabbits treated with ropivacaine alone, there were also no adverse effects on bone healing. Some in vitro studies have demonstrated the cytotoxic potential of local anesthetics, particularly bupivacaine and mepivacaine, and a bupivacaine collagen‐matrix implant delayed bone healing in a rat osteotomy model.^19^, ^20^, ^21^ The lack of effect of ropivacaine on bone healing in the current studies is generally consistent with preclinical and clinical observations for ropivacaine and other local anesthetics, including liposomal bupivacaine, in both bone and wound healing studies.^22^, ^23^, ^24^, ^25^, ^26^, ^27^, ^28^

Interestingly, in rats treated with the mid‐dose of vocacapsaicin, 0.3 mg/kg (0.6 mg/ml), there was a trend for enhanced bone healing relative to control rats, as evidenced by increases in radiographic bone healing scores, percentage of animals with mature or nearly mature calluses, and AUCs (+78%) for the femur in 4‐point bending. Such trends were not observed; however, for the low or high dose in rats or for either dose in rabbits. A potential role of TRPV1 receptors in bone healing has been previously documented in rodent models. For example, in a unilateral open bone (femur) fracture model, TRPV1 knockout mice displayed delayed fracture healing and a reduction in osteoclast and osteoblast differentiation.^29^ In the same model, wild‐type mice displayed increased TRPV1 immunofluorescence during fracture healing.^27^ TRPV1 has also been implicated in the maintenance of structural integrity of intact bone. For example, genetic deletion of TRPV1 or administration of a TRPV1 antagonist protected against ovariectomy‐induced bone loss by inhibiting osteoclast activity.^30^, ^31^ While the potential contribution of vocacapsaicin or other vanilloid‐like agonists to bone healing remains to be elucidated, the trends observed in the present study are consistent with the emerging literature suggesting that TRPV1 receptors are necessary for normal bone healing.

To evaluate the relevance of any potential local or systemic toxicities following vocacapsaicin administration, blood was collected in rabbits for PK evaluation. Across the doses studied, there was a general dose‐dependent increase in systemic exposure for intact vocacapsaicin, CA‐101, and capsaicin. The delivery of vocacapsaicin to the systemic circulation and the production of CA‐101 and capsaicin from vocacapsaicin occurred rapidly. Plasma concentrations were below the limit of quantitation by 2 h for intact vocacapsaicin and by 4 h for CA‐101 and capsaicin. Therefore, systemic exposure to the intact prodrug and its metabolites, including capsaicin, was transient. These findings are consistent with the lack of overt effects of vocacapsaicin on mortality, clinical signs, food consumption, and body weight. Additionally, in single‐ and repeat‐dose toxicology studies conducted in rats and rabbits, where full systemic histopathology and clinical pathology were specifically evaluated, there were no clinically relevant findings and the no‐adverse‐effect level was equal to or higher than the highest concentrations tested in the current study (unpublished data). When vocacapsaicin was administered in combination with ropivacaine, systemic exposure to intact vocacapsaicin, CA‐101, and capsaicin were somewhat lower relative to vocacapsaicin administration alone. Due to the small number of animals tested, it is not possible to ascertain whether this is due to interanimal variability or to a modest effect of ropivacaine on exposure to these analytes.

In summary, a single perioperative administration of vocacapsaicin in rat and rabbit unilateral osteotomy models was well tolerated, locally and systemically, as demonstrated by the absence of mortality directly related to vocacapsaicin, adverse clinical observations, or effect on body weight and food consumption. Systemic exposure to vocacapsaicin and its metabolites, including capsaicin, was transient. In rats, vocacapsaicin was devoid of deleterious effects on bone healing as assessed microscopically and by radiography and biomechanical testing, and there was a trend for enhanced bone healing in rats treated with the mid‐dose, but not low or high doses, of vocacapsaicin. In rabbits, vocacapsaicin was also devoid of adverse effects on bone healing parameters and coadministration of ropivacaine did not alter clinical observations or bone healing findings. The collective findings are consistent with a product that could potentially provide a well‐tolerated, nonopioid, site‐specific treatment for long‐lasting postsurgical pain management.

## CONFLICT OF INTERESTS

Tarra Knotts, Susan Kramer, and John Donovan are employees and shareholders of Concentric Analgesics. Other authors declare no conflict of interest.

## AUTHOR CONTRIBUTIONS

Tarra Knotts, Kirsten Mease, Lakshmi Sangameswaran, and John Donovan contributed to the design, analysis, and interpretation of data, and revising the manuscript. Susan Kramer contributed to the analysis and interpretation of data and revising the manuscript. All authors approved the final version and agree to be accountable for all aspects of the work.

## References

[1] Kehlet H , Dahl JB . The value of “multimodal” or “balanced analgesia” in postoperative pain treatment. Anesth Analg. 1993;77(5):1048‐1056. 8105724 10.1213/00000539-199311000-00030

[2] Wick EC , Grant MC , Wu CL . Postoperative multimodal analgesia pain management with nonopioid analgesics and techniques: a review. JAMA Surg. 2017;152(7):691‐697. 28564673 10.1001/jamasurg.2017.0898

[3] Correll D . Chronic postoperative pain: recent findings in understanding and management. F1000Res. 2017;6:1054. 28713565 10.12688/f1000research.11101.1 PMC5499782

[4] Dumestre DO , Redwood J , Webb CE , Temple‐Oberle C . Enhanced recovery after surgery (ERAS) protocol enables safe same‐day discharge after alloplastic breast reconstruction. Plast Surg. 2017;25(4):249‐254. 10.1177/2292550317728036 PMC5871068 29619347

[5] Lemanu DP , Singh PP , Berridge K , et al. Randomized clinical trial of enhanced recovery versus standard care after laparoscopic sleeve gastrectomy. Br J Surg. 2013;100(4):482‐489. 23339040 10.1002/bjs.9026

[6] Modesitt SC , Sarosiek BM , Trowbridge ER , et al. Enhanced recovery implementation in major gynecologic surgeries: effect of care standardization. Obstet Gynecol. 2016;128(3):457‐466. 27500337 10.1097/AOG.0000000000001555

[7] Gan TJ . Poorly controlled postoperative pain: prevalence, consequences, and prevention. J Pain Res. 2017;10:2287‐2298. 29026331 10.2147/JPR.S144066 PMC5626380

[8] Gan TJ , Habib AS , Miller TE , White W , Apfelbaum JL . Incidence, patient satisfaction, and perceptions of post‐surgical pain: results from a US national survey. Curr Med Res Opin. 2014;30(1):149‐160. 24237004 10.1185/03007995.2013.860019

[9] Caterina MJ , Julius D . The vanilloid receptor: a molecular gateway to the pain pathway. Annu Rev Neurosci. 2001;24:487‐517. 11283319 10.1146/annurev.neuro.24.1.487

[10] Caterina MJ , Schumacher MA , Tominaga M , Rosen TA , Levine JD , Julius D . The capsaicin receptor: a heat‐activated ion channel in the pain pathway. Nature. 1997;389(6653):816‐824. 9349813 10.1038/39807

[11] Tominaga M , Caterina MJ , Malmberg AB , et al. The cloned capsaicin receptor integrates multiple pain‐producing stimuli. Neuron. 1998;21(3):531‐543. 9768840 10.1016/s0896-6273(00)80564-4

[12] Fattori V , Hohmann MS , Rossaneis AC , Pinho‐Ribeiro FA , Verri WA . Capsaicin: current understanding of its mechanisms and therapy of pain and other pre‐clinical and clinical uses. Molecules. 2016;21(7):844. 27367653 10.3390/molecules21070844 PMC6273101

[13] Stella VJ . A case for prodrugs. In: Stella VS , Borchardt R , Hageman M , et al. eds. Prodrugs: Challenges and Rewards. Part 1. 1st ed. Springer; 2007:3‐33.

[14] Gottlieb IJ , Beaton A , Solanki D , et al. A randomized placebo‐controlled trial of intraoperative administration of CA‐008 for post‐operative analgesia following bunionectomy. Poster presented at: 44th Annual Regional Anesthesiology and Acute Pain Meeting; April 11–13, 2019; Las Vegas, NV.

[15] Teichman S , Leiman D , Minkowitz H , et al. Vocacapsaicin reduces pain and opioid consumption for two weeks following a single administration during total knee arthroplasty. Poster presented at: 46th Annual Regional Anesthesiology and Acute Pain Meeting; May 13–15, 2021; Lake Buena Vista, FL.

[16] An YH , Friedman RJ , Draughn RA . Animal models of bone fracture or osteotomy. Animal Models in Orthopaedic Research. CRC Press; 1999:197‐217.

[17] Kramer SM , May JR , Patrick DJ , et al. Instilled or injected purified natural capsaicin has no adverse effects on rat hindlimb sensory‐motor behavior or osteotomy repair. Anesth Analg. 2009;109(1):249‐257. 19535718 10.1213/ane.0b013e3181a7f589

[18] Friel NA , McNickle AG , DeFranco MJ , et al. Effect of highly purified capsaicin on articular cartilage and rotator cuff tendon healing: an in vivo rabbit study. J Orthop Res. 2015;33(12):1854‐1860. 26135547 10.1002/jor.22971

[19] Kreuz PC , Steinwachs M , Angele P . Single‐dose local anesthetics exhibit a type‐, dose‐, and time‐dependent chondrotoxic effect on chondrocytes and cartilage: a systematic review of the current literature. Knee Surg Sports Traumatol Arthrosc. 2018;26(3):819‐830. 28289821 10.1007/s00167-017-4470-5

[20] Breu A , Rosenmeier K , Kujat R , Angele P , Zink W . The cytotoxicity of bupivacaine, ropivacaine, and mepivacaine on human chondrocytes and cartilage. Anesth Analg. 2013;117(2):514‐522. 23749443 10.1213/ANE.0b013e31829481ed

[21] *XARACOLL® full prescribing information*. Innocoll Pharmaceuticals Limited, Athlone, Ireland; 2020.

[22] Henry BJ , Kenison M , McVay C , et al. The effect of local hematoma blocks on early fracture healing. Orthopedics. 2002;25(11):1259‐126. 12452343 10.3928/0147-7447-20021101-17

[23] Baxter R , Bramlett K , Onel E , Daniels S . Impact of local administration of liposome bupivacaine for postsurgical analgesia on wound healing: a review of data from ten prospective, controlled clinical studies. Clin Ther. 2013;35(3):312‐320. 23453403 10.1016/j.clinthera.2013.02.005

[24] Golf M , Daniels SE , Onel E . A phase 3, randomized, placebo‐controlled trial of DepoFoam® bupivacaine (extended‐release bupivacaine local analgesic) in bunionectomy. Adv Ther. 2011;28:776‐788. 21842428 10.1007/s12325-011-0052-y

[25] Smoot JD , Bergese SD , Onel E , Williams HT , Hedden W . The efficacy and safety of DepoFoam® bupivacaine in patients undergoing bilateral, cosmetic, submuscular augmentation mammoplasty: a randomized, double‐blind, active‐control study. Aesthet Surg J. 2012;32:69‐76. 22179931 10.1177/1090820X11430831

[26] Bramlett K , Onel E , Viscusi ER , Jones K . A randomized, double‐blind, dose‐ranging study comparing wound infiltration of DepoFoam bupivacaine, an extended‐release liposomal bupivacaine, to bupivacaine HCl for postsurgical analgesia in total knee arthroplasty. Knee. 2012;19:530‐536. 22285545 10.1016/j.knee.2011.12.004

[27] Davidson EM , Haroutounian S , Kagan L , Naveh M , Aharon A , Ginosar Y . A novel proliposomal ropivacaine oil: pharmacokinetic‐pharmacodynamic studies after subcutaneous administration in pigs. Anesth Analg. 2016;122(5):1663‐1672. 27057797 10.1213/ANE.0000000000001200

[28] Abrão J , Fernandes CR , White PF , et al. Effect of local anesthetic infiltration with bupivacaine and ropivacaine on wound healing: a placebo‐controlled study. Int Wound J. 2014;11(4):379‐85. 23095130 10.1111/j.1742-481X.2012.01101.x PMC7950954

[29] He LH , Liu M , He Y , et al. TRPV1 deletion impaired fracture healing and inhibited osteoclast and osteoblast differentiation. Sci Rep. 2017;7:42385. 28225019 10.1038/srep42385 PMC5320507

[30] Idris AI , Landao‐Bassonga E , Ralston SH . The TRPV1 ion channel antagonist capsazepine inhibits osteoclast and osteoblast differentiation in vitro and ovariectomy induced bone loss in vivo. Bone. 2010;46:1089‐1099. 20096813 10.1016/j.bone.2010.01.368

[31] Rossi F , Bellini G , Torella M , et al. The genetic ablation or pharmacological inhibition of TRPV1 signalling is beneficial for the restoration of quiescent osteoclast activity in ovariectomized mice. Br J Pharmacol. 2014;171(10):2621‐2630. 24308803 10.1111/bph.12542 PMC4009004

